# Epidemiology and clinical characteristics of adult patients presenting to a low resource, tertiary care emergency department in Pakistan: Challenges & Outcomes

**DOI:** 10.12669/pjms.40.2(ICON).8975

**Published:** 2024-01

**Authors:** Wasfa Farooq, Kulsum Kazi, Syed Ghazanfar Saleem, Saima Ali

**Affiliations:** 1 Wasfa Farooq, MBBS, MSc. Pediatric Oncology, Indus Hospital & Health Network, Karachi, Pakistan; 2 Kulsum Kazi, MBBS. Research Department, Indus Hospital & Health Network, Karachi, Pakistan; 3 Syed Ghazanfar Saleem, MBBS, FCPS. Emergency Services, Indus Hospital & Health Network, Karachi, Pakistan; 4 Saima Ali, MBBS, FCPS, MHPE. Emergency Services, Indus Hospital & Health Network, Karachi, Pakistan

**Keywords:** Emergency Care, Emergency Medicine, Clinical Outcomes

## Abstract

**Objectives:**

Emergency Departments (EDs) play a major role in managing acute and chronic illnesses, especially in low-to-middle-income countries like Pakistan, which lacks effective primary healthcare. This study reports the epidemiology and clinical characteristics of patients presenting over a two-year period at the Indus Hospital and Health Network (IHHN) adult ED in Karachi, Pakistan.

**Methods:**

This is a retrospective observational study conducted through chart review of 264,859 patients, aged 16 years and above, who presented to the IHHN ED, Korangi Campus, from January 2019 to December 2020 after obtaining approval from IHHN IRB.

**Results:**

Men were found to be the predominant presenting gender, with a slight rise in the number of women in 2020. The most frequent age group in 2019 was 15-25 (27.7%), whereas it was 25-35 years in 2020 (24.1%), with a decline in total number of elderly visits seen in comparison to previous years. The most frequently seen disposition was ‘referral to clinic’ in 2019 (48.4%) and ‘discharged’ in 2020 (39%). Out of all dispositions, maximum Length of stay (LOS) was seen in patients who left against medical advice in both years. Patient acuity showed the highest number of P3 (moderately ill) patients in both years. Infectious diseases accounted for greater than 10% of patients in both years (17.2% and16.5%), followed by gastrointestinal complaints (15.7% and 11.3%), genitourinary complaints (14.9% and 7.9%), and trauma (11.9% and 12.4%).

**Conclusion:**

Knowledge of epidemiology and clinical characteristics of patients can help facilitate timely planning of staff deployment and allocation of resources to avoid overcrowding, improve patient outcomes, and increase patient satisfaction through timely management.

## INTRODUCTION

Emergency departments (EDs) are often the first point of contact for acute and chronic illnesses in low and middle-income countries (LMIC) due to ill-developed primary health care and insufficient allocation of resources^1^ ED crowding is a global issue, reflecting a need for improvement in emergency care delivery and a deeper understanding of patient demographics, clinical presentations, and necessary interventions.^2^ Studies worldwide have shown diverse ED clinical presentations. Sepsis was the presenting complaint in 61% of adult ED patients in the USA^3^ while breathing difficulties were identified in one third of patients (56/177; 32%).^4^ A study done in Thailand showed head and hip trauma in 20.74%, cardiovascular diseases in 12.93% and respiratory causes in 7.36% as the top three clinical diagnoses in adults visiting the ED.^5^ Similarly, a study done in a tertiary care hospital of Saudi Arabia showed that the frequent visitors to EDs present with gastrointestinal (21.34%), respiratory,^2^ orthopedic, and cardiovascular^3^ complaints.^6^ An African study estimated that up to 24 million lives and nearly one billion Disability Adjusted Life Years (DALY) could be saved by improving healthcare in systems heavily reliant on Eds.^7^

Pakistan lacks a comprehensive national disease prevalence data set, a major challenge in assessing disease burden.^8^,^9^ Unfortunately, the only attempt at an ED-based surveillance system known as the Pak-NEDS pilot study was unable to sustain itself due to budget limitations.^10^ A study done in a public sector hospital in Karachi found high mortality rates in the ED due to treatment gaps, ineffective triage, ED presentation delays and lack of resources.^11^ Data from Karachi, the biggest city of Pakistan, shows that the main causes of adult death occurring in the ED include circulatory disorders and complications of pregnancy,^12^ followed by tuberculosis and trauma from road traffic accidents.^9^

IHHN, based in Pakistan, is a free of cost, philanthropic, health care network with secondary and tertiary centers across 52 districts. Since its inception in 2007, patient data has been captured via an Electronic Health Record system (EHR). IHHN has eleven uniformly modeled EDs^13^ with an annual ED patient visit up to 2.5 million. This gives IHHN the unique opportunity to manage the diverse Pakistani demography, complaints, and outcomes. Previous data collected at IHHN, Karachi showed ED mortality incidence of 0.076% (7.6/10,000 ED visits) in five years.^14^ The Adult Emergency Department at IHHN in Karachi is a 30 bedded facility divided into a fast-track clinic, resuscitation area, major assessment area, rapid assessment area and short stay unit. A total of 16 doctors, including junior and senior physicians, are assigned over two, twelve-hour shifts, with a maximum team of nine physicians in the morning period. An average of 20 nurses are available on each shift.

Using clinical data for the development of healthcare programs and services is considered the foundation of progress and innovation in health.^10^ The onus of collecting data on first patient contact falls primarily on EDs in Pakistan due to lack of holistic primary health care services. High mortality rates and the absence of evidence-based practice in Pakistan have consistently been highlighted and attributed to underutilization and ineffective understanding of locally collected data.^12^,^15^,^16^ Aimed at improving emergency care and efficient usage of ED services in a limited resource setup, this study reports epidemiology and clinical characteristics of patients presenting over a two-year period at the IHHN adult ED in Karachi, Pakistan.

## METHODS

A retrospective observational study was conducted through chart review of patients aged 16 years and above, who presented to the IHHN ED, Korangi Campus, from January 2019 to December 2020. Data were extracted from the Health Management Information System (HMIS) after Institutional Review Board, according to predefined variables. The patient’s ED journey was mapped in three major sections: triage, ED visit and disposition.

### Inclusion criteria

Patients, 16 and above presenting at who presented to the IHHN ED, Korangi Campus, from January 2019 to December 2020.

### Exclusion criteria

Patients, below 16 years of age presenting at the IHHN ED, Korangi Campus

### Ethical approval

Ethical approval was obtained from the Institutional Review Board (IRB) of Interactive Research & Development (IRD) (Study number IRD_IRB_2019_08_001) for retrospective chart review of adult ED data from 2019 and 2020.

### Statistical Analysis

Staffing ratios were calculated by allocation areas and clinical teams in the adult ED. Extracted data was downloaded and stored in a secure database, analyzed for descriptive statistics and cross tabulations for chi square values and visualized graphically using STATA v.13 and RStudio.

## RESULTS

**Fig.1:**
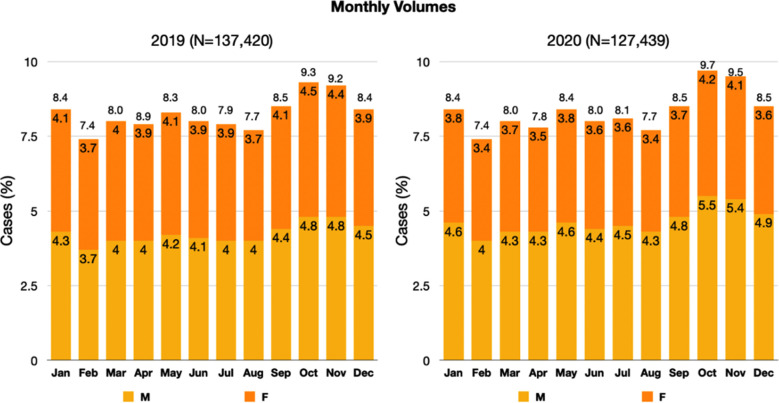
Monthly Trend of Patient Volume, 2019-2020.

A total of 264,859 patients, above 16 years of age, were registered at the adult ED, IHHN Korangi Campus, from January 2019 to December 2020 (Fig.1).

**Fig.2:**
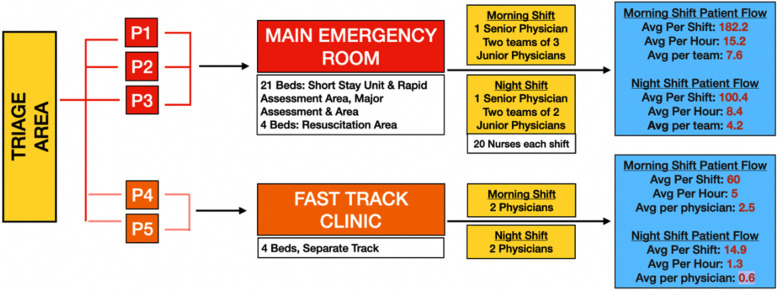
Patient Journey, ED Areas and Average Patient Flow.

Patient flow was tracked from point of registration through triage, where patients’ allocation was done based on acuity (Priority one to five) to either the main ED or the fast-track clinic (Fig.2). Average volumes for each year were calculated to reflect patient presentations per hour, per twelve-hour shift and the average patient number per physician and team.

Of the total patient volume, men were the predominant gender with a slight rise in the number of women in 2020. The most frequent age group in 2019 was 15-25 (27.7%) whereas it was 25-35 years in 2020 (24.1%). Geriatric patients aged 65 years and above accounted for 12,268 (9.6%) in 2019 and 11,668 (8.5%) in 2020. Mean adult age in 2019 was 38.52±16.84 years (median = 35) and 2020 was 39.04±16.63 years (median = 35.07), respectively.

A total of 137,420 patient visits were registered in the adult ED in 2019 by 96,861 patients. Of these patients, 74,747 only made a single visit whereas 22,114(22.8%) patients visited two or more times, with a maximum ED visit value of 37. In 2020, 127,439 patient visits were registered of 86,842 patients, with 65,274(75.2%) patients with one single visit and 21,568 (24.8%) patients visiting two or more times with 47 maximum visits observed. This indicates that of 264,859 total patients’ visits in both years, 183,703 were index visits while 81,156 patients were bounce back visits. This places a huge burden on resource limited EDs in LMIC where lack of structured primary care and out of pocket healthcare expenditure leads to people seeking refuge in EDs for pathologies that can be catered to in a primary care setup.

Patient disposition was divided into six categories; discharged, expired (including dead on arrival), left against medical advice 2 admitted, referred to clinic and referred to other hospitals due to non-availability of bed or relevant specialty. The most frequently seen disposition in IHHN adult ED in 2019 was referral to clinic, seen in 66,526 (48.4%) patients, compared to majority of the patients 49,741(39.0%) who were discharged in 2020. This can be attributed to the ongoing COVID-19 pandemic that resulted in massive volumes of patients presenting to the ED, the majority of whom did not need a follow-up.

**Fig.3:**
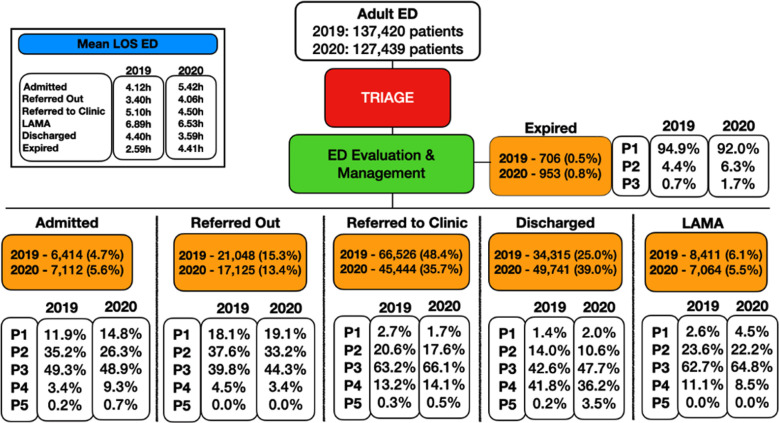
Comparison of disposition with acuity and ED LOS (2019 and 2020).

Patient acuity, assigned according to the Manchester Triaging System (MTS), showed the highest number of P3 (moderately ill) patients in both years, 73,465 (57.6%) in 2019 and 69,460 (50.5%) in 2020, respectively (Figure 2). Mean length of stay 1 was calculated for disposition in both years. Maximum LOS was seen in ‘LAMA’ patients, measuring up to 6.9 hours in 2019 and 6.5 hours in 2020 (Fig.3). This can be explained by an increase in the number of beds in 2020 due to the ongoing COVID-19 pandemic. Increased general distrust of hospitals and healthcare during the pandemic was reflected in patients’ preference to LAMA instead of getting admitted.

The total number of investigations ordered was 496,053 in 2019 and 539,272 in 2020. The maximum number of investigations ordered for a single patient were 51 in 2019 and 84 in 2020, both the patients were MTS acuity P2. This can be explained by the longer LOS of patients due to COVID-19 in 2020 and a larger set of investigative modalities undertaken for diagnosis and management. The average number of tests conducted per patient by acuity in 2019 were 8.3(P1), 6.2(P2), 2.6(P3), 3.8(P4) and 1.9(P5). In 2020, average tests per patient were 11.8(P1), 6.9(P2), 4.6(P3), 3.8(P4) and 2.9(P5). No significant difference in mean investigations per patient were noted in both years as shown in Table-I(a). Disposition was cross tabulated in Table-I(b) and Table-I(c) with gender for both years and mean age per disposition also noted.

**Table-I(a):** Acuity wise Distribution of Investigations.

Acuity	Investigations per patient				

	2019		2020		

N=Frequency	Average tests/patient (SD)	N (%)	Average tests/patient (SD)	N (%)	P value*
P1	8.3 (56.7)	60,442 (12.2%)	11.8 (51.0)	81470 (15.1%)	0.074
P2	6.2 (100.3)	155,096 (31.3%)	6.9 (96.0)	181,100 (33.6%)	0.678
P3	4.6 (132.9)	244,152 (49.2%)	4.6 (137.7)	242,584 (44.9%)	0.711
P4	3.8 (53.9)	36252 (7.2%)	3.8 (57.4)	33,951 (6.3%)	0.689
P5	1.9 (6.6)	111 (0.02%)	2.9 (6.0)	167 (0.03%)	0.561

**Table-I(b):** Cross-tabulation of Gender with Disposition including mean age (2019).

2019 N=Frequency	Male N (%)	Mean Age, Y (SD)	Female N (%)	Mean Age, Y (SD)	Total N (%)
Admitted	2,014(3.5)	47.24 (18.5)	4,402(7.1)	34.04 (15.7)	6,416(5.4)
Referred out	11,748(20.4)	44.49 (18.9)	9,300(15.1)	46.26 (18.6)	21,048(17.6)
Referred to Clinic	29,361(50.9)	39.45 (17.0)	37,163(60.2)	36.66 (15.2)	66,524(55.7)
Discharged	9,177(15.9)	34.55 (15.4)	7,131(11.5)	35.92 (15.0)	16,308(13.7)
LAMA	4,917(8.5)	35.75 (16.0)	3,494(5.6)	37.40 (15.8)	8,411(7.0)
Expired	414(0.8)	55.77 (18.1)	292(0.5)	55.92 (17.4)	706(0.6)
Total	57,631	39.22 (17.4)	59,782	37.83 (16.2)	119,413

**Table-I(c):** Cross-tabulation of Gender with Disposition including mean age (2020).

2020 N=Frequency	Male N(%)	Mean Age, Y (SD)	Female N(%)	Mean Age, Y (SD)	Total N(%)
Admitted	2,348(4.7)	49.07 (18.6)	4,764(9.3)	34.34 (15.8)	7,112(7.1)
Referred out	9,828(19.9)	45.12 (18.7)	7,297(14.3)	46.65 (18.4)	17,125(17.1)
Referred to Clinic	19,054(38.6)	40.54 (16.9)	26,390(51.8)	36.24 (14.8)	45,444(45.3)
Discharged	13,598(27.5)	36.36 (14.9)	9,062(17.8)	36.22 (14.8)	22,660(22.5)
LAMA	4,003(8.1)	37.82 (16.9)	3,061(6.0)	39.35 (16.7)	7,064(7.0)
Expired	572(1.2)	58.00 (16.7)	381(0.8)	54.72 (17.7)	953(1.0)
Total	49,403	40.03 (16.6)	50,955	37.92 (16.0)	100,358

**Fig.4:**
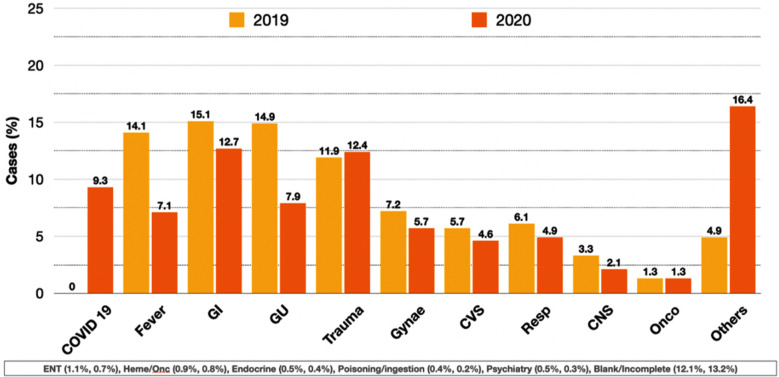
ED Diagnosis by complaint category.

The primary complaints of patients presenting to the Adult ED were manually sorted according to categories and frequencies as seen in Fig.4. Gastrointestinal complaints accounted for greater than 10% of patients in both years (15.1% and 12.7%), followed by genitourinary complaints (14.9% and 7.9%), fever (14.1%, 7.1%) and trauma (11.9% and 12.4%). COVID-19 patients were kept in a separate area at the onset of the pandemic, accounting for a total of 9.3% patients in 2020. The category of ‘Other’ included non-specific complaints such as pain, weakness as well as those patients who had been triaged for IV medications and post-operative follow ups. This number is much higher in 2020, 16.4%, as due to the coronavirus pandemic, a greater number of patients were diverted to the ED in place of the consulting clinic and elective admission.

## DISCUSSION

ED quality of service is globally compromised by high patient volumes. This constant demand of service causes a shortfall in resources, a more pressing challenge in LMICs.^1^ The situation is worsened when the approach towards efficient service is not based on contextual data and actual demographics of the catchment population. Local Pakistani studies with the aim to develop strategies for efficient management and timely off-loading of EDs is the need of the day to meet this challenge effectively.

Data analysis in our study showed a prevalence of men in 2019 and 2020 (53% and 51% respectively). Similar findings were seen in a Saudi context where men were three times more likely to be in the frequent users’ groups than women and in a study from South India where men (64%) were the prevalent gender as compared to women (36%).^6^,^17^ Gender was found to be a strong effect modifier indicating that the combined effects of gender and chronic disease are multiplicative. Men with one to two chronic diseases had 1.58 times odds of frequent ED visit when compared to men with no chronic diseases. Likewise, men with three to five chronic diseases had 4.98 times odds of frequent ED use when compared to those with no comorbidities.^17^

The highest patient age frequency seen in our ED was between 15-25 years in 2019 (27.7%) and 25-35 years in 2020 (24.1%). This age distribution of frequent ED visits reflects the population pyramid in Pakistan, with most of the population being young adults.^18^ On the other hand, geriatric patients aged 65 years and above accounted for 12,268 (9.6%) of patients presenting in 2019 and 11,668 (8.5%) in 2020. Previous data collected from a tertiary care setup in Karachi showed that almost 24% (n = 13014) of all adults (n = 54588) presenting to the ED were over the age of 60 years.^19^ Before the outbreak of COVID-19, patients with age 60–69 years had the most frequent ED visits (45.42%) in Thailand with pneumonia (4.8%), malaise and fatigue (4.5%), and heart failure (4.3%) seen as common pathologies.^5^,^20^

As shown in Fig.2, the average patient flow per team in the main ED was 7.6 patients in the morning shift and 4.2 in the night shift. Division between individual physicians in the morning shift makes the per physician hourly figure 2.5 patients in the morning and 0.6 patients at night. Bed availability and physician staffing are intricately connected factors to overcrowding,^21^ the observed average of 2.5 patients per physician per hour is indicative of overcrowding in our setting. Delayed patient assessment and care provision as a result of overcrowding in the ED could result in increased mortality, medical error, and decreased patient satisfaction.^22^ Additionally, this average does not matter since physicians present in our ED are often untrained medical officers placing additional burden on the available senior emergency medicine physician.

Acuity was cross tabulated with disposition and mean LOS for each disposition in both years, showing that most patients were referred to the clinic or discharged. This could be attributed to critical patients being referred out from the ED after initial stabilization due to unavailability of beds and lack of certain medical and surgical service lines. Out of all dispositions, maximum LOS was seen in LAMA patients in both years, 6.9 and 6.5 hours respectively. Major reasons might include reluctance of the family for transfer to an unfamiliar hospital resulting in forceful stays, and refusal by other hospitals for further management due to their lack of facilities.^23^ In most cases, Paul et al. found out that the frequency and causes for LAMA vary according to the ailment, geographical region, and type of healthcare system.^24^ All of these problems were worsened by the COVID-19 pandemic.

The most common complaints in this two-year data, accounting for greater than 10% of patients in both years, were gastrointestinal complaints (15.1%, 12.7%), followed by genitourinary complaints (14.9%, 7.9%), fever (14.1%, 7.1%) and trauma (11.9%, 12.4%). Karachi, like other megacities, is majorly plagued by infectious diseases.^25^ High population density, coupled with lack of clean drinking water and sanitation for masses, makes it extremely susceptible, particularly amongst the underserved population, the main cohort seen at IHHN, Karachi. Out of various epidemics seen in Karachi, allergy/cough, asthma/flu and fever/skin rashes appear as prominent groups, with the peak season seen in January and October, followed by bronchitis, malaria, diarrhea, rhinitis, sinusitis and typhoid as dominating epidemics seen in various densely populated, poor hygiene areas of Karachi.^4^,^26^

Previous studies have demonstrated that elderly ED patients are of a higher acuity with 24.8% presenting with life threatening conditions requiring resuscitative measures as compared to 13.8% in younger groups.^19^ Data from the US has demonstrated 24.9% - 28.9% high acuity visits among the elderly in studies by Nawar, Wolinsky and others.^19^ Results from our study showed that the highest number of patients presented as acuity P3 according to MTS in both years, 73,465 (57.6%) in 2019 and 69,460 (50.5%) in 2020 respectively. A similar trend was seen in the elderly population (>65 years) with 40.6% and 41.8% of P3 patients respectively in 2019 and 2020.

The fall in the number of geriatric patients in our study is reflective of a study from Finland showing a decline in total number of ED visits by 16% during the COVID-19 pandemic lockdown.^27^ Similar results were seen in Milan, Italy with approximately 50% decline during March and April 2020, as compared with the same months in 2019.^20^ The marked decline in ED visits of vulnerable elderly population can perhaps be attributed to the effect of lockdown measures and fear of contagion from medical facilities.^28^ Necessity of social distancing precautions for older adults may have further isolated and disconnected them from families and caregivers who may have been the primary point of contact for health concerns, including transportation to the ED.^26^

Most of the patients presenting to IHHN are young daily wage earners, including people travelling from outer cities with none to meager means of accommodation within Karachi, with a wish to return home once basic healthcare has been given. Literature cites refusal of procedure/operation (23.2%), long ED waiting time (19.8%), subjective improvement with treatment (17.7%) and children at home (14.8%) as the most common reasons for LAMA.^29^ Unfortunately, our study was unable to capture reasons for the high frequency of LAMA in our sample population.

In line with a previous study conducted at IHHN over a span of four years with mortality rates of 0.4% observed in 2014-2018,^14^ mortality of 0.5% and 0.9% was noted in 2019 and 2020. The most common ED diagnoses at death in 2019 were poisoning, cardiopulmonary arrest and dead-on-arrival in whom the cause of death could not be ascertained. In 2020, only 8.4% of total mortalities were due to suspected or severe COVID-19, with cardiopulmonary arrest remaining the most frequently seen cause of death.

A noteworthy upsurge was seen in the number of investigations ordered between 2019 and 2020. This can be attributed to the increase in the number of beds in 2020 and a longer mean LOS for patients as compared to 2019. As the highest acuity presenting to the ED was P3, it can be observed that in 2020 more patients were discharged, referred to clinic or referred out than were admitted in midst of the COVID-19 peak.

Overcrowding in the ED leads to delay in patient care, an unpleasant reality for patients.^23^ Visiting an ED for low-acuity conditions is often motivated by convenience and health anxiety triggered by time constraints and focused usage of multidisciplinary medical care in a highly equipped setting.^30^ An essential role of primary health care physicians in this regard is still missing in the medical landscape of Pakistan. The use of this data set can be multiplicative in mapping the patient disease burden per month or per season, such as seasonal dengue or influenza outbreaks. This allows for better care and guidance, preparation for a large influx and efficient patient management. More effective tools, with Artificial Intelligence and modelling, can be designed to predict emergency department workload and improve resource planning.

### Limitations

Despite IHHN containing digital records through EMR services, integrated data including each area of patient care was not easily extractable. Due to the voluminous patient records, ICD coding of the ED data was not available, and records were open text. This resulted in manual sorting of data from HMIS for patient complaints and ED diagnosis. Detailed comparison of patients, their presentations and diagnoses were not possible. Physician appropriateness, reasons for LAMA and patient satisfaction are also missing from the current study.

## CONCLUSION

With limited primary health care services in Pakistan, annual ED patient visits at IHHN reach up to an overwhelming number of 2.5 million per year. Accurate prediction of patient attendance and clinical characteristics can help facilitate timely planning of staff deployment and allocation of resources to avoid overcrowding, improve patient outcome and increase patient satisfaction by timely management.

### Authors’ contributions:

**WF:** Prepared tables, figures and wrote the methods and results section of the manuscript. **KK**: Prepared a significant part of the manuscript text and reviewed for accuracy. **SA:** Prepared a significant part of the manuscript text and reviewed for accuracy. **SGS** conceptualized the article, worked on data analysis and reviewed the manuscript text for overall accuracy and integrity. All authors reviewed the manuscript.
